# Inflammatory biomarkers at different stages of Sarcopenia in older women

**DOI:** 10.1038/s41598-023-37229-3

**Published:** 2023-06-26

**Authors:** Leonardo Augusto da Costa Teixeira, Nubia Carelli Pereira Avelar, Marco Fabrício Dias Peixoto, Adriana Netto Parentoni, Jousielle Marcia dos Santos, Fabiana Souza Máximo Pereira, Ana Lúcia Danielewicz, Amanda Aparecida Oliveira Leopoldino, Sabrina Paula Costa, Arthur Nascimento Arrieiro, Luana Aparecida Soares, Vanessa Kelly da Silva Lage, Ana Caroline Negreiro Prates, Redha Taiar, Alessandra de Carvalho Bastone, Vinicius Cunha de Oliveira, Murilo Xavier Oliveira, Henrique Silveira Costa, Juliana Nogueira Pontes Nobre, Franciane Pereira Brant, Tamiris Campos Duarte, Pedro Henrique Scheidt Figueiredo, Vanessa Amaral Mendonça, Ana Cristina Rodrigues Lacerda

**Affiliations:** 1 Programa de Pós-Graduação em Ciências da Saúde (PPGCS), Universidade Federal dos Vales do Jequitinhonha e Mucuri, Diamantina, MG Brazil; 2 Departamento de Fisioterapia da Universidade Federal de Santa Catarina (UFSC), Campus Aranguá, Santa Catarina, Brazil; 3 Programa de Pós-Graduação em Reabilitação e Desempenho Funcional (PPGReab), Universidade Federal dos Vales do Jequitinhonha e Mucuri, Teófilo Otoni, MG Brazil; 4 Departamento de Fisioterapia, Faculdade de Ciências Biológicas e da Saúde, Universidade Federal dos Vales do Jequitinhonha e Mucuri, Diamantina, MG Brazil; 5 Faculdade de Ciências Médicas de Minas Gerais (FCMMG), Programa de Pós-Graduação em Ciências da Saúde, Belo Horizonte, MG Brazil; 6 MATériaux et Ingénierie Mécanique (MATIM), Université de Reims Champagne-Ardenne, 51100 Reims, France

**Keywords:** Cytokines, Diagnostic markers, Predictive markers, Endocrine system and metabolic diseases, Biomarkers, Epidemiology, Biomarkers, Diseases, Endocrinology, Medical research

## Abstract

In recent years, studies have found that Sarcopenia alters inflammatory biomarkers. However, the behavior of inflammatory biomarkers at different stages of Sarcopenia is not well understood. This study aimed to compare a broad panel of inflammatory biomarkers in older women at different stages of Sarcopenia. The study included 71 Brazilian community-dwelling older women. Muscle Strength was assessed by using handgrip strength (Jamar dynamometer). The Short Physical Performance Battery (SPPB) was performed to assess the physical performance, and body composition was assessed by DEXA. Sarcopenia was diagnosed and classified according to the EWGSOP2 criteria. Blood was drawn, and inflammatory biomarkers associated with Sarcopenia (IL-2, IL-4, IL-5, IL-6, IL-8, IL-10, TNF, adiponectin, leptin, resistin, BDNF, sTNFr-1 and sTNFr-2) was analysed. After diagnosis and classification of sarcopenia, 45% of women did not present Sarcopenia (NS, N = 32), 23.9% were diagnosed with Sarcopenia Probable (SP, N = 17), 19,7% with Sarcopenia Confirmed (SC, N = 14), and 11.3% with Severe Sarcopenia (SS, N = 8). The analysis of inflammatory biomarkers revealed that the more advanced the stage of Sarcopenia, the higher the levels of BDNF, IL-8, sTNFr-1, and sTNFr-2. The assessment of BDNF, IL-8, sTNFr-1, and sTNFr-2 levels may be an adjuvant tool in diagnosis and severity classification of Sarcopenia in older Brazilian women.

## Introduction

According to the International Classification of Diseases (ICD-10-MC-M62.84), Sarcopenia is a syndrome characterized by progressive loss of muscular mass, muscle strength, and physical function that increases risk of falls, hospitalization and mortality^1–3^.

Sarcopenia affects older people globally to a range of 10–27%^4^, while in Brazil it ranges from 1.8%^5^ to 49.2%^6^. This variation is caused by the use of different instruments for assessing muscle mass and strength, distinct cut-off points for each global population, and different classification algorithms^4^. Evidence indicates the feminization of aging in Brazil^7^, increasing the rates of Sarcopenia in older women. Advanced age, cognitive impairment, lower income, smoking, and malnutrition are potential risk factors for the incidence and severity of Sarcopenia^8^. In 2010, the European Study Group on Sarcopenia (EWGSOP1) presented classification criteria for the three stages of Sarcopenia: pre-sarcopenia, Sarcopenia, and severe Sarcopenia^9^. However, in 2018 the EWGSOP revised the guidelines. The diagnostic process consisted of three steps: (1) screening for low muscle strength to identify individuals with probable Sarcopenia; (2) identifying low muscle mass to confirm the presence of Sarcopenia; and (3) identifying low physical performance to assess the severity of Sarcopenia^1^. Thus, the classification was stratified into non Sarcopenia, probable Sarcopenia, confirmed Sarcopenia, and severe Sarcopenia.

Sarcopenia is also characterized by endocrine, inflammatory, and metabolic disorders, and the balance of pro- and anti-inflammatory biomarkers regulates muscle regeneration mechanisms playing a critical role in determining the disease severity^10–12^. A pro-inflammatory profile is frequently associated with a reduction in muscle mass, strength, mobility, and physical performance in sarcopenic individuals^3,11,12^.

The measurement of clinical biomarkers is faster and less expensive than the direct measurement of the final clinical outcome for sarcopenia diagnosis^13^. Biomarkers are generally used to screen, diagnose, characterize, and monitor diseases^13^. However, the potential biomarkers involved in the pathogenesis of Sarcopenia have been the topic of of intense debate^3,10–12^. Thus, the development of a panel of inflammatory biomarkers to assist in the diagnosis and severity classification of Sarcopenia has been a major area of interest^3^. Adiponectin, Leptin, Resistin, Interleukin (IL)-6, IL-8, IL-10, tumoral necrosis factor (TNF), soluble receptor of TNF (sTNFr)-1 and sTNFr-2 have been reported as potential inflammatory biomarkers associated with the diagnosis of Sarcopenia, and an imbalance between pro- and anti-inflammatory cytocines was associated with loss of mass and muscle function and locomotor disabilities^10–13^. Brain-derived neurotrophic factor (BDNF) has also been identified as a possible biomarker related to sacopenia^14,15^. The presence of high concentrations of pro-inflammatory markers are related to greater systemic and skeletal muscle inflammation, indicative of the presence and severity of sarcopenia^10–15^.

However, a panel of biomarkers that characterize the diagnosis and severity of sarcopenia has yet to be studied^10,11^. The identification of biomarkers that describe Sarcopenia is challenging due to the disease's complexity and multifactorial pathogenesis^10,16^. In this context, the purpose of this study was to compare a panel of inflammatory biomarkers among older women with different stagies of Sarcopenia.

## Methods

### Study design

This is an exploratory, observational, and cross-sectional study. The study was approved by the Research Ethics Committee of the Universidade Federal dos Vales do Jequitinhonha e Mucuri (UFVJM) (protocol 1.461.306). All participants signed a written Informed consent. All evaluations were carried out between June 2016 and June 2017 at the Laboratório de Fisiologia do Exerccio (LAFIEX) and Laboratório de Inflamaço e Metabolismo (LIM) from the UFVJM.

### Participants

Older women from the community were considered for the present study. A survey was carried out of the total number of older women (> 65 years) registered in all primary care units in the city of Diamantina, Minas Gerais, Brazil. All addresses were visited to invite participants. All participants who accepted the invitation answered a clinical questionaire. The inclusion criteria were women over the age of 65 who were functionally independent in the community and capable of completing the study evaluations. The exclusion criteria was subjects who had comproved cognitive disfunction in the Mini Mental State Examination (MMSE)^14^; those with neurological sequelae; those who were hospitalized less than 3 months ago; who had fractures in the lower or upper limbs for less than 6 months; who had acute musculoskeletal disorders that interfered with the proposed physical assessments; who had acute respiratory or cardiovascular diseases; that they could not perform the respiratory measures maneuvers; who had an inflammatory disease in the acute phase; neoplasm in activity in the last 5 years; in palliative care; who were using anti-inflammatory medications or drugs that act on the immune system; and those with significant visual or auditory deficits that would make it impossible to carry out the proposed procedures.

### Procedures

The evaluations were carried out during three laboratory visits on three different days. On the first visit, participants who met the eligibility criteria signed the written informed consent, and answered the clinical health interviews. On the second visit, participants underwent body composition measurements in the morning while fasting from food, beverages, and medications. After a 15-min pause for rest, all participants performed the handgrip strength and physical performance tests. On the third visit (24 h after physical tests), participants’blood samples were drawn for analysis of blood inflammatory biomarkers.

### Assessment of muscle strength

Handgrip strength (HGS) was evaluated using a Jamar dynamometer. The participant was instructed to maintain a seated position with a neutral hand, flexed elbow, and neutral shoulder. The HGS measurement, i.e., an isometric contraction the dominant hand applied on the handles of the dynamometer, was expressed in kilogram-force (kgf). The average of three measurements was used for the analysis^15^. The cutoff point adopted was < 20 kgf for women established by physical function for diagnosis of Sarcopenia in a longitudinal study on aging^1,16^.

### Assessment of body composition

Body composition was measured using Dual X-ray Absorptiometry (DXA Lunar Type DPX—encore software 2005), one of the gold standard method for assessing muscle mass in Sarcopenia^17,18^. Body composition measurements were conducted in the morning by the same researcher (08:00 a.m.). Skeletal muscle mass index (SMI) was calculated by the ALM/height squared ratio (ALM/h^2^), the cutoff point used for low SMI was < 5.5 kg/m^2^^1,9^.

### Assessment of physical performance

The Short Physical Performance Battery (SPPB) test was used to evaluate the physical capacity of participants with respect to their balance, gait speed, and lower limb muscular strength^19^. The SPPB is composed of tests of static balance while standing, walking speed at the usual pace, and muscle strength of the lower limbs estimated by the sit-to-stand test from an armless chair. A final score of 12 points is calculated for each test based on the performance indicated by scores ranging from 0 to 4 (respectively, the worse and better performance). Participants who obtained scores from 0 to 3—had a poor performance or had inability; from 4 to 6—had low performance; from 7 to 9—had moderate performance; from 10 to 12—had good performance^19^. The cutoff point for low physical performance was ≤ 8 points^1,9^.

### Sarcopenia stages classifications

Sarcopenia was diagnosed by performing the evaluations according to the EWGSOP2 guidelines, beginning with muscle strength, to identify older women with probable Sarcopenia^1^. To confirm Sarcopenia and determine severe Sarcopenia, we utilized the classification criteria proposed by the EWGSOP1^9^, which proposes the diagnosis based on the documentation of criterion 1, plus criterion 2 or criterion 3 to confirm Sarcopenia, and the presence of all three criteria to confirm severe Sarcopenia. Therefore, the participants were classified into four groups: (1) non-sarcopenia (NS)—Those who did not have loss in muscle strength, in muscle mass and physical performance; (2) sarcopenia probable (SP)—Those who had only loss of muscle strength; (3) Sarcopenia Confirmed (SC)—Those who had loss of muscle strength together with loss of muscle mass or low physical performance; and (4) Severe Sarcopenia (SS)—Those who had loss of muscle strength, muscle mass and low physical performance concomitantly.

### Analysis of blood inflammatory biomarker

Blood was drawn at 8 a.m. (10 mL from the antecubital fossa of the upper limb with disposable material) after participants fasted from food and drink and without using medication for 10 h. The samples were drawn in vacutainer bottles with heparin in a sterile environment. Immediately after this procedure, the samples were centrifuged at 3000 rpm in a centrifuge for 10 min. Plasma samples were extracted and kept at − 80 °C for 6 months before being analyzed. The IL-2, IL-4, IL-5, IL-10, TNF, adiponectin, leptin, resistin, BDNF, sTNFr-1 and sTNFr-2 levels were analyzed by Enzyme-linked Immunosorbent technique (ELISA) (Duo-Set, R&D Systems, Minneapolis, USA). The plasma levels of IL-6, IL-8, and IFN were measured using the cytometric bead arrays kit (BD Bioscience, San Jose, CA) according to the manufacturer’s protocol. Samples were acquired in a FACSCanto flow cytometer (BD Bioscience) and analyzed using the FCAP array v1.0.1 software (Soft Flow)^20^.

### Statistical analyses

Openepi Software (www.openepi.com) was used to determine the sample size, taking into account a population of 2522 older women (> 65 years old) in the municipality of Diamantina, Minas Gerais, as registered by the Brazilian Institute of Geography and Statistics (IBGE—ibge.gov.br). Thus, considering a sarcopenia prevalence of 16%^7^, an effect size of 0.80, a significance level of 5%, and a confidence interval of 80%, a sample size of 71 older women were met. The Statistical Package for the Social Sciences (SPSS Statistics, version 22.0, IBM, Armonk, NY, USA) and the Med-Calc Statistical (Med-Calc Software, version 13.1, Ostend, Belgium) softwares were used for statistical analyses. Data normality was verified by the Kolmogorov–Smirnov test. Continuous variables were expressed as the minimum and maximum values and median. The one-way ANOVA test was used for comparison between groups when the variables were parametric, and the Kruskal Wallis test for independent samples was used for comparison between groups when the variables were non-parametric.

### Institutional review board statement

The study will be conducted according to the guidelines of the Declaration of Helsinki and was approved by the Institutional Ethics and Research Committee of Federal University of Jequitinhonha and Mucuri Valleys (protocol code 1.461.306 on 22 March of 2016).

### Informed consent

Informed consent will be collected from all subjects involved in the study.

## Results

**Figure 1:**
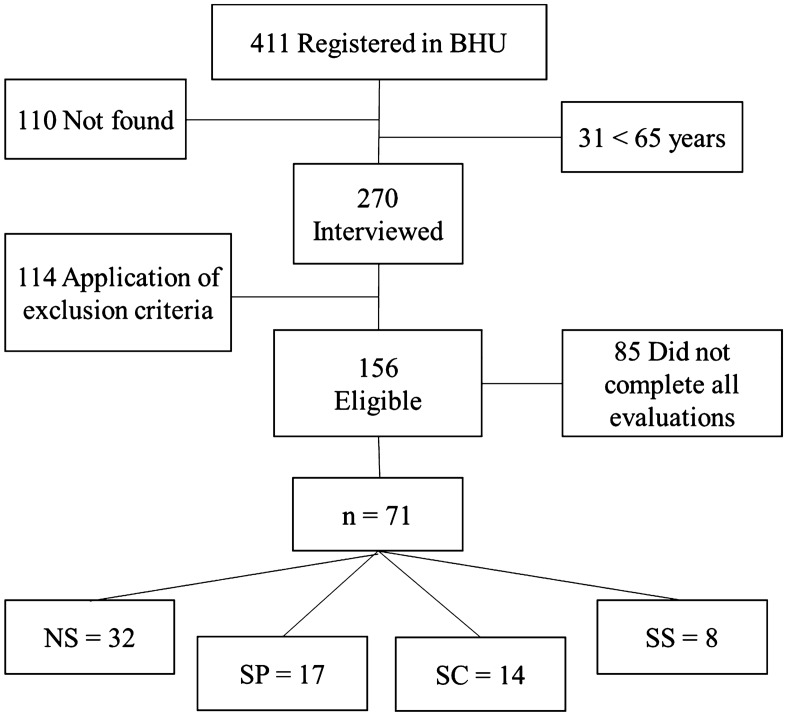
Flowchart of the study’s participants. *BHU* basic health units, *NS* non-sarcopenia, *SP* sarcopenia probable, *SC* sarcopenia confirmed, *SS* sarcopenia severe.

A total of 411 older women were identified based on their BHU registration. One hundred and ten addresses were not found, and thirty-one were excluded since they did not meet the study's age criteria inclusion.Two hundred seventy women were interviewed, and a total of 114 were excluded based on the exclusion criteria. One hundred fifty-six women living in the community were eligible for the study. Eighty-five older women did not complete all evaluations; thus, the sample size was composed of 71 women. After the evaluations, we found 32 women without Sarcopenia, 17 with probable Sarcopenia, 14 with verified Sarcopenia, and 8 with severe Sarcopenia after the evaluations (Fig. 1).

The diagnosis of probable sarcopenia (SP) confirmed Sarcopenia (SC) and severe Sarcopenia (SS) was found in 23.9%, 19.7% and 11.3% respectively. Participants had a mean age of 75 years old (± 7), a mean height of 1.50 m (± 0.05), a mean muscle mass index (SMI) of 6.39 kg/m^2^ (± 1.05), a mean handgrip strength (HGS) of 19.9 kgf (± 6.38), and a mean of 8.62 points (± 2.01) in the SPPB test. Blood inflammatory biomarker data revealed an increase in the ratio of pro inflammatory biomarkers as the severity of Sarcopenia increased (Table 1).

**Table 1:** Characteristics of the participants according to the sarcopenia severity (*n* = 71).

Variable	Sarcopenia stages								
	Non-sarcopenia (*n* = 32)		Probable sarcopenia (*n* = 17)		Sarcopenia (*n* = 14)		Severe sarcopenia (*n* = 8)		p value
	Min–max	Median	Min–max	Median	Min–max	Median	Min–max	Median	
Age	66–96	71.5	65–88	76	67–94	78	66–88	77.5	0.16
Height (m)	1.44–1.63	1.52	1.39–1.57	1.46	1.40–1.55	1.46	1.45–1.61	1.50	0.002^a,b^
Total fat mass (kg)	13.11–36.40	25.48	12.34–37.81	21.93	9.27–33.95	18.69	11.23–25.37	16.46	0.002^b,c^
Total lean mass (kg)	19.19–47.87	37.38	27.25–37.66	32.92	25.88–37.54	31.51	22.87–35.66	28.37	< 0.001^a,b,c^
HGS (kgf)	20.33–40.00	24.63	8.66–19.66	16.33	11.66–20.00	17.16	4.00–20.00	15.66	< 0.001^a,b,c^
SMI (kg/m^2^)	4.86–9.63	7.05	5.57–7.91	6.23	5.23–7.70	5.71	4.12–5.58	4.90	< 0.001^b,c,d^
SPPB	3–12	9	7–12	10	5–11	8.5	2–8	7	0.001^c,d^
Adiponectin (µg/mL)	20.2–62.8	49.5	36.15–57.45	50.02	47.98–57.42	51.03	38.37–56.26	50.82	0.69
IFN (pg/mL)	0.84–2.35	1.38	1.06–1.87	1.31	0.95–14.01	1.47	1.06–1.51	1.31	0.478
IL-2 (pg/mL)	3.52–5.16	4.06	3.59–5.16	4.14	3.67–5.83	4.1	3.67–4.81	3.98	0.706
IL-4 (pg/mL)	1.67–2.4	2.02	1.74–2.57	1.92	1.67–28.17	2.04	1.89–2.28	2.12	0.611
IL-5 (pg/mL)	0.50–8.59	0.72	0.59–3.16	0.71	0.47–6.67	0.80	0.60–2.00	0.90	0.292
IL-6 (pg/mL)	10.89–22.69	16.33	12.61–25.48	17.24	9.20–35.72	18.04	13.43–22.97	16.49	0.412
IL-10 (pg/mL)	1.10–8.99	1.60	1.14–1.91	1.58	1.28–29.06	1.65	1.23–1.63	1.55	0.416
Leptin (µg/mL)	1.45–2.22	1.96	0.84–2.20	1.81	1.31–2.31	1.90	1.43–2.05	1.87	0.500
Resistin (µg/mL)	0.74–2.24	1.63	0.99–2.09	1.58	1.00–2.38	1.79	1.19–2.15	1.73	0.616
TNF (pg/mL)	0.84–1.6	1.08	0.75–1.36	1.08	0.84–16.85	1.13	0.88–1.36	1.05	0.958

*HGS* handgrip strength, *SMI* skeletal muscle mass index, *SPPB* short physical performance battery, *BDNF* brain derived neurotrophic factor, *TNF* tumor necrosis factor, *sTNFr* soluble receptor of TNF, *INF* interferon, *IL* interleukin.

Data express in minimum and maximum values and median.

^a^Difference between NS and SP groups.

^b^Difference between NS and SC groups.

^c^Difference between NS and SS groups.

^d^Difference between SP and SS groups. (^abcd^ = p < 0.05).

**Figure 2:**
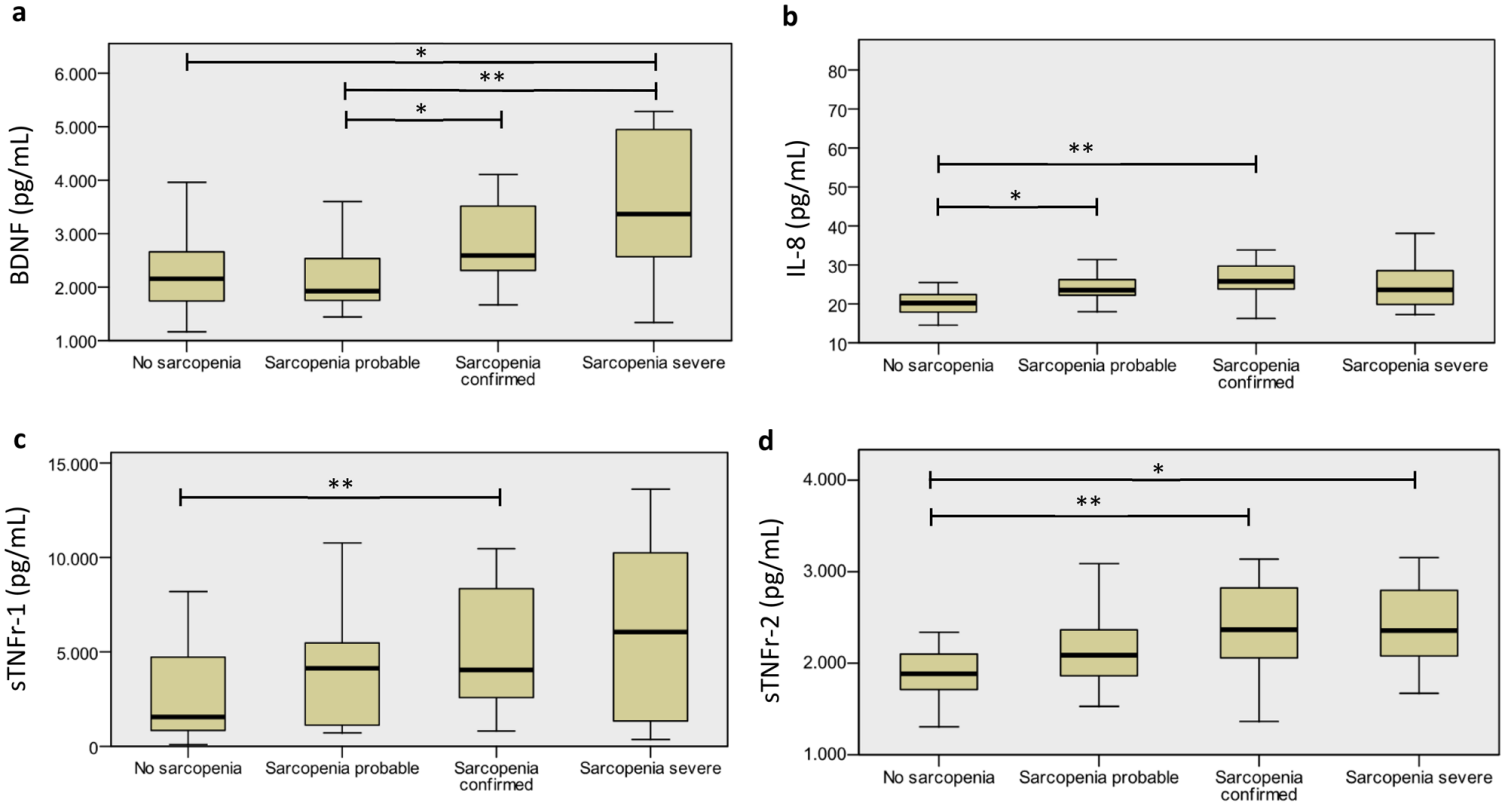
Distribution of biomarkers between groups. Plasma concentrations of biomarkers are higher in groups with more severity of sarcopenia. Comparisons were made using the ANOVA test and kruskal wallis depending on the distribution of the variable. In (**a**) plasma concentrations of BDNF (pg/ml). (**b**) IL-8 (pg/ml). (**c**) sTNFr-1 (pg/ml). (**d**) sTNFr-2 (pg/ml). Blood inflammatory biomarkers of the participants according to the sarcopenia severity (*n* = 71). *BDNF* brain derived neurothophic factor, *TNF* tumor necrosis factor, *sTNFr* soluble receptor of TNF, *IL-8* interleukin 8; *difference statistically significant using Kruskal Wallis test for independent samples (*p < 0.05 and **p < 0.01).

Analysis of the distribution of biomarkers showed significant differences in the plasmatic concentrations of BDNF, IL-8, sTNFr-1 and sTNFr-2 between all groups (Fig. 2).

## Discussion

To our knowledge, this is the first study to investigate a broad panel of inflammatory biomarkers according to the severity of Sarcopenia in older women. The major finding of this study is that when the severity of Sarcopenia increases in older Brazilian women, an imbalance in inflammatory biomarkers occurs that favors a pro-inflammatory state. Specifically, we found higher levels of BDNF, IL-8, sTNFr-1 and sTNFr-2 as the sarcopenia severity increased.

As expected, body composition results revealed lower levels of muscle mass and strength, as well as physical performance, as the severity of Sarcopenia increased^1,2,4,9,21,22^. Interestingly, there was no difference in age between the groups, indicating that the severity of sarcopenia can occur regardless of more advanced age. In addition, no differences were found in the height among SS and NS groups, indicating that height is not a determinant factor for the diagnosis of severe sarcopenia.

The increased levels of IL-8 in the SP and SS groups compared to the NS group indicate that Sarcopenia induces a pro-inflammatory state. Interleukin-8 (IL-8) is a chemotactic factor that promotes inflammation^12^, and high plasma levels of this biomarker were associated with less strength gain during resistance training, less lean body mass, and a greater risk of Sarcopenia^11,12^. In line with these results, IL-8 levels increased as the severity of Sarcopenia increased among the participants of this study. The differences were observed in two situations: among NS and SP groups, and among NS and SC groups. Within sarcopenia subgroups there was no significate difference in the concentration of this marker i.e., no significant differences were found in IL-8 among the SP and SC or SS groups. In addition, there was no significant difference in IL-8 levels between NS and SS groups. These results might indicate that IL-8 increases in the early stages of Sarcopenia.

The levels of IL-8, sTNFr-1, and sTNFr-2 differed among the groups with confirmed Sarcopenia, and these differences probably contribute to the impaired muscular strength in these groups. In a previous study, we demonstrated that sTNFr-1 and sTNFr-2 are predictors of the functional performance of individuals with chronic diseases, with high levels of these biomarkers correlated to poor performance^23^. Other studies have reported that sTNFr-1 initiates the inflammatory response and stimulates realease IL-6^24^ and others pro- and anti-inflammatory cytokines^6,24,25^.

Schaap et al. found a negative correlation between sTNFr-1 and a 5-year change in thigh muscle area in the older^25^. Similarly, Gonzalo-Calvo et al. found that high levels of sTNFr-1 are associated with functional dependency in the older population^26^. However, opposite results were found by Lustosa et al., who investigated the association between muscular strength and blood sTNFr-1 levels in 63 community-dwelling older women divided into non-sarcopenic (n = 32) and sarcopenic (n = 31) groups^6^. Using the same EWGSOP1 algorithm, tools, and procedures as in the present investigation, they found that sarcopenic older women had lower muscle strength and blood sTNFr-1 levels than non-sarcopenic older women (p = 0.01). However, as Lustosa et al. categorized the sample into only two groups, they were unable to examine sTNFr-1 levels at different stages of Sarcopenia. According to our findings, the levels of sTNFr-1 increased as the severity of Sarcopenia increased; nevertheless, we identified statistically significant differences only between the non-sarcopenic and sarcopenic groups.

Recently, sTNFr-2 levels were positively correlated with serum progranulin levels, a key marker of frailty; however, sTNFr-2 levels were not associated with Sarcopenia (assessed by SARC-F)^27^. Other studies found that reduction in the sTNFr-2 levels and were associated with improvements in health conditions^28^ and physical performance in individuals with chronic diseases^23^. In the current investigation, as Sarcopenia is more severe, sTNFr-2 levels were higher, and statistically significant differences were identified between the NS and SC and SS groups. These results indicate a pivotal role of sTNFr-2 in the inflammatory responses of Sarcopenia, especially when muscle strength and mass, as well as physical performance, reduce.

Brain-derived neurotrophic factor (BDNF) is a neurotrophin involved with neuronal growth, differentiation, and plasticity^29^. In skeletal muscle BDNF is involved with the development and differentiation of myoblasts into muscle fibers, motoneuron survival and transmission synaptic^30^. BDNF is produced in skeletal muscle cells especially during muscle contractions or in response to injury^31^, acting in an autocrine or paracrine manner, and playing an important role in muscle repair^30^. Increased levels of BDNF have also been reported in severe illnesses characterized by high levels of systemic inflammation^31^. Similarly, our findings also revealed a possible association between BDNF levels and the severity of Sarcopenia. Interestingly, the most significant differences were observed between sarcopenic groups, suggesting the involvement of BDNF in the severity of Sarcopenia.

BDNF has been found to play a critical role in regulating neuromuscular function during aging in a mouse model^32^. On the other hand, other studies found that frailty older Korean and Japanese presented low levels of BDNF and was negatively associated with physical performance and severity of Sarcopenia^29^. Serum BDNF levels were positively correlated with quadriceps femoris thickness (= 0.096, p = 0.006), however there was no association between BDNF and handgrip strength (= 0.046, p = 0.197) or walking frequency. Interestingly, participants who required assistance to stand from their chairs had significantly (p = 0.028) lower serum BDNF levels than those who stood without assistance^34^. Importantly, the measuring instruments were imprecise, and questionnaires were used to evaluate crucial physical and functional characteristics of the study participants^34^. In this investigation, we employed the methodologies, tools, and algorithm closest to the recognized gold standard for the evaluation of Sarcopenia.

It should be emphasized that BDNF levels differed across all stages of Sarcopenia, demonstrating a correlation between the progression of Sarcopenia and BDNF production in community-dwelling older women. There is evidence that BDNF expression increases in response to muscle injury, and inflammation can stimulate the generation of high levels of BDNF^30,31,35^. This enables us to hypothesize that BDNF is implicated in the physiopathology of Sarcopenia and that its rise is related to muscle injury and impaired neuromuscular synaptic transmission caused by Sarcopenia and its progression. Further study along these lines is required to elucidate the pathophysiological mechanism underlying the association between BDNF and sarcopenia severity.

Using the same population, studies comparing EWGSOP1 with EWGSOP2 guidelines found substantial differences in prevalence, clinical outcomes, and risk factors^36,37^. Thus the order of screening and assessment can result in different outcomes found. Recently, Sayer and Cruz–Jentoft published a commentary suggesting that muscle mass levels are a common element for the diagnosis of various health conditions, including sarcopenia^2^.

Inflammatory biomarkers that indicate muscle catabolism are highly correlated with frailty in multimorbid patients^38^, and previous studies have associated adiponectin levels with reduced appendicular muscle mass independent of body fat in older women^39^. It is crucial to consider the algorithm employed in our sample for evaluating screening procedures based on handgrip strength, being muscle mass or physical performance is a secondary component in determining Sarcopenia. Thus, there may exist distinct and specific biomarkers that indicate low muscle strength, muscle mass, and physical function, as well as when these losses occur concomitantly. Further studies are needed to determine whether or not there is a biomarker capable of distinguishing the loss of strength, muscle mass, and physical performance in sarcopenic older individuals.

We highlight that the sample analyzed in this study was homogeneous, thereby reducing the influence of clinical, sociodemographic, cultural, functional, or lifestyle characteristics. The present study should be interpreted with caution due to the short duration of observation (cross-sectional design), the limited number of older women evaluated in each group, and the absence of correlation or association analyses. Nevertheless, even with a small sample size, statistically significant differences were observed, and we used the methodologies, instruments, and algorithm closest to the gold standard methods for sarcopenia evaluation. In addition, other strengths of the present study were the investigation of a large panel of biomarkers previously associated with Sarcopenia, the blinding of the researchers in the evaluations, the methodological rigor, and the tight inclusion and exclusion criteria of the study.

Our study provides information on the behavior of several inflammatory biomarkers according to the severity of Sarcopenia in older women and indicates a worsen in phisical performance and a pro-inflamatory state as sarcopenia increases in severity.

In clinical terms, our study describes the behavior of several inflammatory biomarkers accordinf to the severity of Sarcopenia and reveals a decline in physical performance and a pro-inflammatory state as the severity of sarcopenia increases in older women. This study's findings may also be valuable for clinicians and researchers seeking a more comprehensive understanding of the diagnosis and severity of sarcopenia.

## Conclusion

Taken together, the results of this study reveal for the first time that the greater the severity of Sarcopenia in older women, the higher the blood levels of BDNF, sTNFr1, sTNFr2, and IL-8. These findings may improve the comprehension of the diagnosis and severity of Sarcopenia in older women.

## Data Availability

The data presented in this study are available on request from the corresponding author. The data are not publicly available due to the privacy guarantee of the data collected individually.
